# Retired medical simulation manikins as a development platform for physically assistive robots: a staged roadmap

**DOI:** 10.3389/frobt.2026.1933962

**Published:** 2026-09-10

**Authors:** Nabil Zary, Jalal Alfroukh, Mohamed Alali

**Affiliations:** Institute of Learning, Mohammed Bin Rashid University of Medicine and Health Sciences, Dubai Health, Dubai, United Arab Emirates

**Keywords:** healthcare robotics, human-centered design, human-robot interaction, manikin repurposing, medical simulation manikins, physically assistive robots

## Abstract

Physically assistive robots require body designs that ensure safe contact, build trust, and are cost-effective for healthcare providers. However, dedicated platforms often struggle to achieve all three simultaneously. We suggest that high-fidelity medical simulation manikins could serve as a practical development platform in this field. These manikins have an anthropomorphic shape with human-like contact surfaces, internal space for routing cables and mounting devices, and are familiar to clinical staff. Because simulation programs frequently replace their functional units, this hardware can be deployed directly within the institutions that need assistance. Based on a scoping review of 64 studies on manikin adaptation and a pilot project that transformed a retired manikin into a multilingual receptionist for about US$1,200 in additional parts, we propose a staged roadmap: social and cognitive interaction, sensory enhancement, guided physical contact, and active physical assistance, with measurable stage gates governing each transition. The pilot demonstrates only feasibility for conversational and embedded computing, not physical assistance or user acceptance. We recognize limitations, including the need for new load paths, certification under ISO 13482 and related standards, and prospective ethics approval in later stages. Additionally, the realistic appearance of the manikin may pose risks, as added motion to a static, humanlike form can cause discomfort. This roadmap aims to turn these uncertainties into testable hypotheses using an affordable platform and to encourage the assistive robotics community to view the simulation storeroom as a valuable resource.

## 1 Introduction

Healthcare systems face a widening gap between the need for physical assistance, such as help with daily activities, mobility, rehabilitation, and transfers, and the available workforce. Physically assistive robots offer a promising solution, with the development of capable platforms for personal care, manipulation, and support (Mukai et al., 2010; Erickson et al., 2020; Kyrarini et al., 2021). This robot class must meet three key demands: first, robots that interact physically with people must have body shapes and contact surfaces designed for safe and comfortable human-robot interaction (HRI) (Haddadin and Croft, 2016). Second, users must trust these machines enough to accept physical contact, a level of trust that customized machinery does not automatically establish (Broadbent, 2017). Lastly, the organizations that most need these robots often operate on tight budgets, while such assistive platforms can cost from tens of thousands to hundreds of thousands of dollars.

A neighboring field has devoted nearly 60 years to creating artifacts designed for human touch. The earliest computer-controlled patient simulator, Denson and Abrahamson’s 1969 Sim One, marks an important beginning: it was technologically innovative but failed commercially due to cost, unlike the accessible and simple Resusci Anne, which gained global popularity. Since then, the simulation industry has advanced to produce high-fidelity manikins with realistic body proportions, flexible coverings, articulated limbs, and embedded sensors, sufficiently refined for clinicians to treat them as actual patients. As simulation technology evolves rapidly, institutions often replace functioning manikins with newer versions. Although both fields are interested in realistic embodiment and convincing interactions, collaboration between the simulation and assistive robotics communities remains limited (Zary et al., 2025).

This Perspective suggests that retired manikins can serve as development platforms for physically assistive robots and outlines a staged roadmap for their advancement. The principal contribution is the translational roadmap itself: a staged, gated pathway toward physical assistance. Two subsidiary claims support it. The first is a reuse strategy for retired institutional assets and an affordable entry point for embodied HRI research; we keep these distinct because they require different evidence. Throughout, we mark each claim as demonstrated (shown by the Badr pilot), inferred (supported by published work from other groups), or hypothesized (requiring future testing). The argument rests on two empirical sources from our team, both currently in preprint: a scoping review of 64 studies on manikin adaptation for robotic capabilities (Zary et al., 2025), and Badr, a pilot project in which a retired manikin was transformed into a functioning receptionist at a health sciences facility (Alali and Zary, 2025). Because these sources have not yet undergone peer review, we present their quantitative findings as preliminary and ground each roadmap stage in independent, published evidence where available. The proposed roadmap addresses key open challenges in this area, including human-centered interface design, customizable physical interfaces, trust and safety perceptions in pHRI, and hospital deployment.

## 2 What manikins offer, described honestly

Manikins offer four essential benefits for assistive robot development, and each is bounded in ways that matter for the roadmap.

An anthropomorphic envelope. High-fidelity manikins emulate human proportions, joint placement, and surface geometry at a level rarely achieved by purpose-built robots. For a robot interacting with humans, this envelope is practical: contact surfaces are already designed for human contact, and the compliant covering minimizes stiffness at contact. It does not serve as a load path. Manikin skeletal structures are friction-jointed positioning armatures intended for chest compressions and transport, not for supporting or transmitting forces. Training manikins are not designed to stand or balance without support. Any stage of the development process that encounters significant loads must provide its own supporting structure, with the manikin offering only the envelope and surface features.

Internal volume and integration features. Manikins include routing channels, mounting points, and space for electronics. Their built-in sensors, such as compression-depth sensors and airway switches, are narrow in scope, task-specific, and confined to proprietary vendor systems. Consequently, repurposing typically requires replacing these sensors. Badr did not reuse any of the original sensing components. This vendor lock-in underscores the importance of developing repurposed platforms with open middleware from the outset.

Institutional familiarity. Manikins are common in healthcare settings and are easily handled by staff who have used them for years. They are stored and procured through established channels, and when a manikin is modified and introduced into a hospital, it feels like familiar equipment rather than a strange device. However, this familiarity does not imply suitability for all contexts. Manikins are designed to enable trained clinicians to suspend disbelief during limited training sessions that involve a different population, consent process, and emotional environment than those encountered when unwell individuals receive care. Some patients even find manikin faces unsettling. While staff familiarity is demonstrated by decades of simulation practice, whether care recipients accept them remains a hypothesis our platform aims to test.

A fleet is available, but with limitations. Institutions typically retire manikins on a regular cycle, primarily because they degrade over time. Coverings become tacky or brittle, pneumatics develop perforations, and electronics lose vendor support. Some are traded in, stripped for parts, or refurbished to preserve resale value. The realistic pool of candidates includes units retired for outdated features rather than physical deterioration. Any program should begin with a condition assessment, marked as Stage 0 in the roadmap below, using explicit and reproducible exclusion criteria (Table 1). The total global pool size is unknown. Conducting an inventory of retired simulation assets would be a valuable early initiative for this community.

**TABLE 1:** Stage gates for the manikin repurposing roadmap. Each stage lists entry criteria, measurable exit criteria that gate progression to the next stage, and safety requirements. At Stage 4, the original manikin contributes the anthropomorphic envelope, contact surfaces, and the interaction stack matured in earlier stages; structure, joints, and actuation are new.

Stage	Entry criteria	Exit criteria (gate to next stage)	Safety and fail-safe requirements
0. Condition triage	Retired unit available with known service history	Structure sound under rated static load; covering intact and decontaminated; proprietary electronics removable; provenance documented. Exclude units with structural damage, degraded or contaminated coverings, or undocumented modification	Bench assessment only
1. Social and cognitive interaction	Stage 0 passed	Dialogue task success and language coverage targets met; response latency within conversational norms; sustained unattended reliability over weeks	No contact by design; power loss returns the system to an inert state
2. Sensory augmentation	Stage 1 stable in deployment	User state estimation validated against reference measures; sensing accuracy, latency, and failure detection meet predefined thresholds; thermal budget verified in the sealed envelope	Contactless by design; degraded sensing triggers safe shutdown
3a. Non-weight-bearing contact	Stage 2 gates passed; prospective ethics approval for contact studies	Contact force and pressure within thresholds derived from ISO/TS 15066, adjusted for the user group; compliant actuation characterized; emergency release verified	Non-weight-bearing by design; supervised sessions; release to passive state on power or control loss
3b. Balance cueing and steadying contact	Stage 3a gates passed; clinical risk analysis under ISO 14971	Stability margins verified under worst-case perturbation; allowable support loads defined and tested; fall risk controls validated under supervision	Locked or fixed base; trained supervisor present; defined power-loss behavior; audible state signaling
4. Active physical assistance	New load-bearing chassis; ISO 13482 conformity strategy; IEC 80601-2-78 where clinically deployed	Certified load transfer at rated payload; validated adaptive control; longitudinal acceptance evidence in the target population	Full personal care robot safety case; continuous clinical oversight

Our review of 64 studies from 1969 to 2024 shows that efforts to enhance manikins with robotic features are advancing along three main avenues (Zary et al., 2025): hardware enhancements, shifting from basic sensor setups to fully actuated systems; software advancements, evolving from rule-based systems to biomimetic control; and hybrid approaches that integrate manikins with mixed reality, teleoperation, and haptic feedback (Samosky et al., 2012). These developments are primarily limited to training contexts, with little integration into broader robotics systems. Only four of 64 studies employed the Robot Operating System (Macenski et al., 2022).

## 3 The badr pilot

To assess whether a retired manikin can function as a deployed robot, our team created Badr, a high-fidelity adult manikin converted into a receptionist at the Institute of Learning, part of a multi-department academic health system in Dubai (Alali and Zary, 2025). Because the original report is a preprint, we summarize the key points here.

The manikin was modified to support a distributed computing setup: a Raspberry Pi four manages hardware interfaces, motion detection, and orchestration, while a Raspberry Pi five equipped with an AI accelerator handles more intensive perception and language tasks. It includes a camera, audio input and output, and a passive infrared sensor, all governed by a tiered activation scheme to reduce power consumption between interactions. The system can communicate in English and Arabic, including the Emirati dialect, provide departmental information, and alert staff to arrivals. The additional component cost was approximately US$1,200, significantly lower than the US$15,000 to US$30,000 typically needed for commercial humanoid receptionist systems. Studies have shown that embodied receptionists foster stronger engagement than screen-based options (Bazzano and Lamberti, 2018).

Badr was conducted as an internal service pilot and is reported accordingly. The basis for that designation is explicit: the pilot involved no research procedures with human participants, and no participant dataset was collected. Images, audio, identifiers, and usage metrics were not retained for research purposes; processing served immediate operational needs under internal institutional oversight. It confirms technical feasibility: a retired manikin operated as a functioning robot in a real reception setting, amid variable noise, lighting, and traffic. It shows that meaningful AI capabilities can run on low-cost embedded hardware when processing is appropriately distributed. What it does not demonstrate is equally important; it provides evidence of operational uptime rather than user acceptance. No user study was conducted, and social acceptance of an information kiosk among passing visitors does not imply willingness to be touched, as trust in physical contact differs from social approval (Broadbent, 2017). It does not include actuation, force sensing, or any physical assistance. In the roadmap, Badr provides evidence only for Stage 1.

Two architectural lessons carry over to later stages. Separating hardware control from AI processing prepares the control structure required for actuated stages, and the layered software design accommodates new hardware interface layers for actuators and force sensors without requiring restructuring.

## 4 A staged roadmap with measurable gates

**FIGURE 1:**
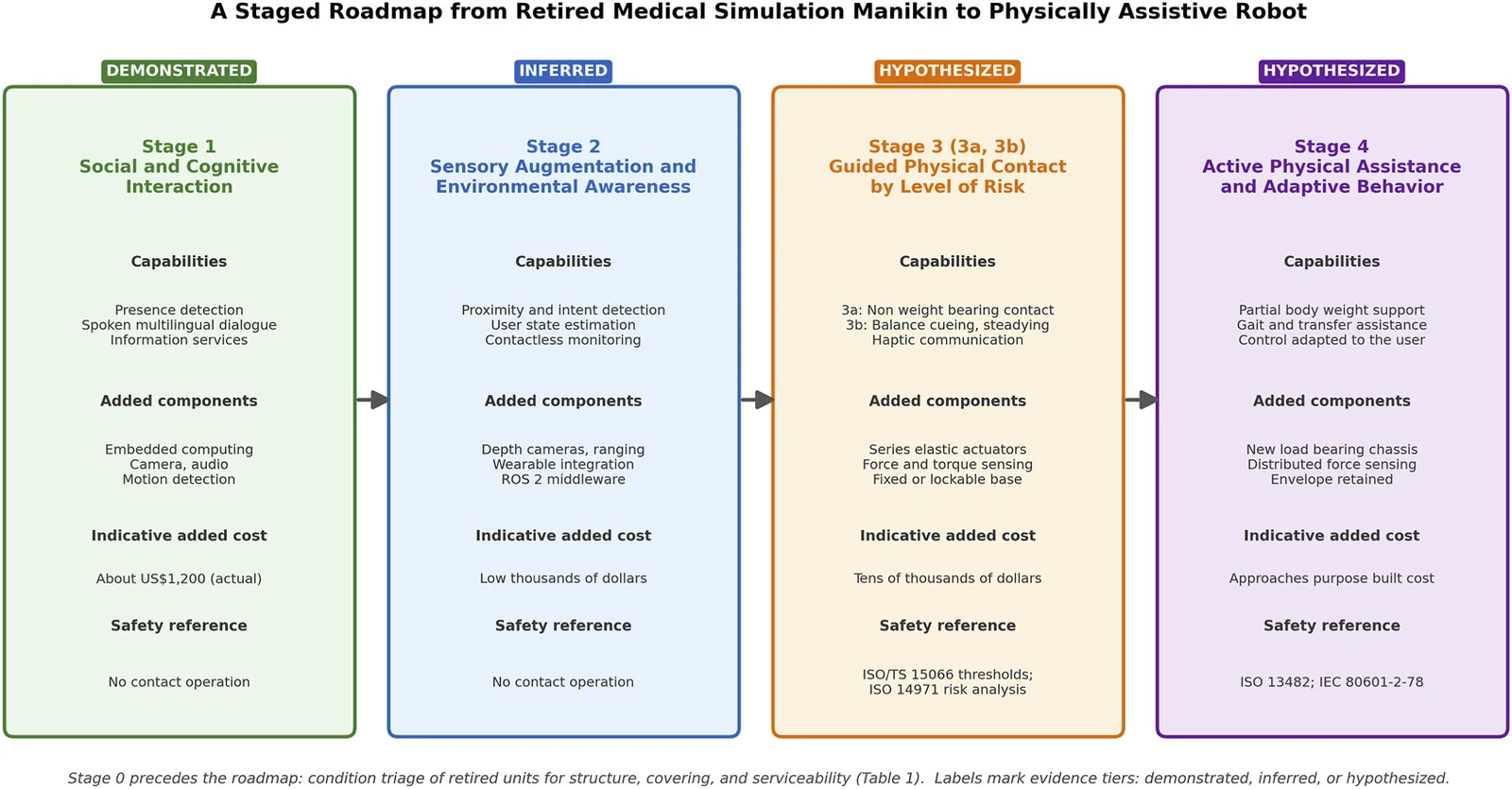
A staged roadmap from a retired medical simulation manikin to a physically assistive robot. A preliminary condition triage (Stage 0) screens retired units. Four stages then progress from social and cognitive interaction, demonstrated by the Badr pilot, through sensory augmentation and guided physical contact to active physical assistance, with capabilities, added components, indicative added cost, and applicable safety references identified at each stage. Stage gates are defined in Table 1.

Figure 1 illustrates the roadmap, and Table 1 defines its gates: entry criteria, measurable exit criteria, and safety requirements that enable an independent team to decide when a platform may advance. A preliminary Stage 0, known as condition triage, evaluates retired units for structural integrity, condition, and serviceability before any investment is made. Exclusion criteria include structural damage, degraded or contaminated coverings, undocumented modifications, and unsupported proprietary electronics (Table 1). Each subsequent stage enhances a functioning platform, maintains previous capabilities, and allows an institution to halt at the stage that best fits its needs.

Stage 1 (demonstrated) involves social and cognitive interactions, including presence detection, spoken dialogue, multilingual communication, and information services. Badr demonstrates this at an additional cost of about US$1,200 for components. This stage is fundamental to physical assistance: users must first trust and understand the robot. The language skills developed here enable the robot to announce and explain physical actions before making contact. This layer advances quickly as large language models become more integrated with robotic perception and actions (Ahn et al., 2022).

Stage 2 (inferred) focuses on sensory augmentation and environmental awareness. It involves contactless detection of proximity and gestures, recognition of user intent, and estimation of physical states such as posture, gait stability, and fatigue. This is achieved with added hardware, including depth cameras, ranging sensors, optional wearable integrations, and enhanced edge computing, at costs in the low-thousands-of-dollars range. Key engineering constraints include ensuring that sensing equipment fits within the manikin without affecting its appearance and managing the thermal budget within the sealed shell for the compute and power units. Moving forward, the platform should operate on ROS 2, linking modified manikins to common navigation, manipulation, and safety tools (Macenski et al., 2022) and addressing the lack of standard middleware in this field.

Stage 3 (hypothesized) involves guided physical contact, which we categorize based on physical risk levels because haptic signaling and steadying support have different implications when there is a potential for loss of power, control, or stability, leading to a fall. Stage 3a includes non-weight-bearing contact such as haptic signaling, guided reaching, and touch communication, where failure does not pose a fall risk, and the platform becomes passive if power or control is lost. Stage 3b pertains to balance cueing and steadying contact during standing, where a user might briefly load the platform. This stage requires confirmed stability margins under worst-case disturbances, defined support load limits, supervised operation, emergency release options, and specific behaviors in case of power loss. The setup includes a fixed or lockable wheeled base positioned beside a bed or chair because the manikin itself lacks a stable stance or balance. Weight-bearing support belongs to Stage 4, not Stage 3. Hardware enhancements include compliant actuation, such as series elastic actuators (Pratt and Williamson, 1995), force and torque sensors at contact points, and mechanical joint limiters, with an estimated cost in the tens of thousands of dollars. Pneumatic muscle alternatives require compressors and valves, which the device cannot accommodate. The applicable standards must be evaluated on a per-use-case basis. ISO/TS 15066 (ISO, 2016) was written for industrial collaborative robots; it provides useful starting thresholds for contact force and pressure at Stage 3a, but it is insufficient on its own for contact with older adults or clinically vulnerable users, whose tissue tolerance and fall consequences differ. ISO 13482 (ISO, 2014) frames the personal care robot safety case from Stage 3b onward, and clinical deployment at any contact stage also requires risk management under ISO 14971 (ISO, 2019), supervision protocols, and clinical risk controls beyond any robotics standard. Contact surfaces that come into contact with patients should be made of disinfectant-tolerant medical-grade silicone, because original manikin skins tolerate only mild cleaning agents and are damaged by typical hospital disinfectants.

Stage 4 (hypothesized) involves active physical assistance and adaptive behavior, including partial body-weight support, gait aid, transfer assistance, and individualized control (Hogan, 1985). This stage requires replacing the manikin structure with a specialized load-bearing chassis while maintaining the previous envelope and interaction stack. At that point, the platform is best described as an assistive robot wearing the manikin envelope: what remains of the original unit is the anthropomorphic shell, the contact surfaces, and the interaction stack, which matured in earlier stages, while the structure, joints, and actuation are new (Table 1). The reference points highlight the complexity: RIBA, a dedicated patient-lifting robot, requires high-stiffness arms and a wide-wheeled base to lift a person (Mukai et al., 2010). Similarly, the Panasonic Resyone robotic care bed, the first device certified under ISO 13482, illustrates the engineering and certification challenges posed by regulatory standards for physical assistance. At this stage, devices are governed by ISO 13482 for personal care robots (ISO, 2014) and by IEC 80601-2-78 for rehabilitation and assessment robots (IEC, 2019).

Regarding regulation, proper claims must be made for each stage. Certification is tied to specific intended uses, so each contact escalation counts as a new intended use that requires assessment; approval does not transfer between stages. The real benefit of staging lies in early deployment advantages, gradually reducing engineering risks, and collecting evidence at low stakes before the high-stakes design phase. In addition, repurposing alters liability: the original manufacturer did not design, warrant, or document these platforms for assistance roles, so the deploying organization assumes that responsibility.

The core economic argument remains valid despite these adjustments. The $1,200 figure applies only to Stage 1 and cannot support affordability claims for later stages. A realistic assessment rests on the total cost of ownership: inspection, decontamination or covering replacement, structural modification, actuation and sensing, engineering labor, maintenance, software support, safety testing, certification, liability, and disposal, with comparators that are functionally equivalent at each stage, from commercial reception systems at Stage 1 to certified care robots at Stage 4. Repurposing remains favorable because the envelope, integration provisions, and institutional familiarity offset the cost of purchasing equivalent anthropomorphic hardware; it becomes unfavorable when structural replacement dominates the cost, which is why Stage 4 approaches purpose-built territory. The sustainability benefits of reuse are expected rather than demonstrated and should be established with lifecycle evidence. Institutions start with an existing asset, gain value from Stage 1 onward, and invest heavily only after the low-cost stages resolve the acceptance questions that the costly stages depend on.

## 5 Human factors, hypotheses, and deployment

The main uncertainty in this roadmap is human-related rather than mechanical, and the platform’s realism influences this risk. A static human-like form that begins to move is near the worst part of the uncanny valley, as motion tends to heighten the sense of eeriness (Mori et al., 2012). In addition, anthropomorphism is a highly variable design choice that can evoke either comfort or threat, depending on the context (Broadbent, 2017). A recent systematic review and meta-analysis of AI-based human-robot interaction (HRI) in clinical healthcare settings highlights measurable impacts on patient trust, communication, engagement, and bonding. However, it also warns that the variability among studies and reporting limitations necessitate cautious interpretation of the combined results (Shafaie et al., 2026). These outcome areas are suitable dependent variables for this program, with a consistent boundary: evidence of social or contactless acceptance should not be mistaken for a willingness to accept physical contact.

The staged plan aims to assess this risk in advance. It includes three hypotheses. First, repurposed manikins are expected to score similarly to functionally matched non-anthropomorphic platforms on safety and trust among care recipients. Second, perceived eeriness is expected to increase from the static Stage 1 to the moving Stage 3 platform, but design features such as announced movement are expected to mitigate this increase. Third, prior social interactions at Stage 1 are predicted to increase willingness to engage in physical contact at Stage 3. Investigating these hypotheses requires more than just questionnaires. It is essential to differentiate between clinical staff, who are familiar with manikins, and care recipients, who lack such familiarity. The exposure order should be randomized and repeated to separate effects of novelty and sequence from consistent preferences. Primary outcomes should involve validated scales like the Godspeed and trust-in-automation tools (Bartneck et al., 2009), combined with behavioral endpoints such as observed consent and completion of a contact task, along with measures of perceived control and dignity. Regardless of the results, findings from this affordable hardware can inform design across the field, including applications involving remote body surrogates, where embodiment issues often arise (Grice and Kemp, 2019).

Customization claims must accurately reflect the available options. Manikins are offered in adult, pediatric, and obstetric models to showcase some morphological variety. Yet, existing simulation literature reveals noticeable gaps: default units often depict light-skinned, lean, adult male figures. These disparities also appear in platforms that have been adapted for different uses. In our deployment environment, additional factors are crucial, including ensuring the platform’s gender presentation aligns with cross-gender care norms and features that resemble the local population’s phenotypes. Incorporating language support, such as Badr’s Arabic functionalities, addresses the software side of fulfilling these customization requirements.

Implementing deployment in institutions raises governance issues that must be addressed early, with requirements increasing at each stage. The platform class includes features like identifying individual users and managing personal and biometric data, while later phases involve engaging with users who might have limited ability to give consent. A governance framework should align with the technical development: clear notice and data minimization in Stages 1 and 2; ethics approval, individual consent, and privacy measures for any contact study at Stage 3; and at Stage 4, consent processes and dignity protocols tailored for users with limited capacity, validated infection control for replaced coverings, and ongoing clinical supervision. Any formal human subjects research, operational use of personal identification, or patient interactions from Stage 3 onward will require ethics approval in advance; this is considered a necessary milestone on the roadmap.

## 6 Concluding remarks

This Perspective makes a deliberately modest yet valuable claim. Physically assistive robots require human-compatible design, acceptance, and economical development options. Retired simulation mannequins effectively address the first need, enable inexpensive testing for acceptance, and enhance the third by allowing organizations to leverage existing assets. However, they do not offer load-bearing capacity, certified safety, or guaranteed acceptance; these aspects are addressed through specific stages, milestones, and evidence tiers in the roadmap. The shell, storage, and familiarity within institutions are real and sufficient to justify this approach.

The significant challenges of Stages 3 and 4, such as compliant actuation within an anthropomorphic envelope, certified contact safety, and adaptive control, remain unresolved frontiers for the entire field. Although these issues are not simplified when using a manikin, they become more affordable to tackle, enabling earlier answers to key questions about trust, comfort, and dignity. Realizing this vision demands collaboration among robotics engineers, simulation technologists, rehabilitation clinicians, and human factors researchers. A practical initial step is to inventory retired simulation assets in healthcare institutions. We encourage this community to explore the simulation storeroom, as it offers a valuable platform for development.

## Data Availability

The raw data supporting the conclusions of this article will be made available by the authors, without undue reservation.
